# Effect of administrative information on visit rate of frequent attenders in primary health care: ten-year follow-up study

**DOI:** 10.1186/s12875-018-0836-0

**Published:** 2018-08-28

**Authors:** Anne K. Santalahti, Tero J. Vahlberg, Sinikka H. Luutonen, Päivi T. Rautava

**Affiliations:** 1 Turku City Healthcare Center, Turku, Finland; 2 Department of Biostatistics, Faculty of Medicine, University of Turku, Turku, Finland; 3 Department of Psychiatry, University of Turku, Turku, Finland; 4 Department of Psychiatry, Turku University Hospital, Turku, Finland; 5 Department of Public Health, Faculty of Medicine, University of Turku, Turku, Finland; 6 Turku Clinical Research Centre, Turku University Hospital, Turku, Finland

**Keywords:** Primary care, Frequent attenders, Administrative information, GP’s work load

## Abstract

**Background:**

Frequent attenders (FAs) use a disproportionately large share of the resources of general practitioners (GPs) working in primary healthcare centres. The aim of this study was to estimate the proportion of FAs among all patients in the primary health care centres of a medium-sized city in Finland, and to examine whether providing GPs with administrative information about their frequent attenders (names and numbers of visits per year) can reduce the number of FAs and the frequency of their visits.

**Methods:**

Statistic data on all GP visits (*n* = 1.8 million) to 11 public healthcare centres in one city were collected from the electronic patient records covering the period from 2001 to 2010. A FA-patient was defined as a person who made10 or more visits to GPs during one year. The baseline situation in 2001 was compared with the situation in 2006 after administrative information had been provided three times to all GPs working in the healthcare centres. Poisson’s regression analysis was used, and FA numbers and consultation rates in the years 2002–2005 were compared with the year 2006; figures for 2006 were also compared with those for the follow-up period 2007–2010.

**Results:**

During the years 2001–2006, the proportion of visits of FA-patients fell overall from 9.1 to 8.5%, a decline of 0.6% (*p* < 0.0001). This reduction was equivalent to an annual work load of two GPs in the study center. The proportion of visits of FA patients increased again in the follow-up period (2007–2010), when administrative information was no longer provided.

**Conclusion:**

When GPs are provided with information on the number and names of their FA-patients, the annual rate of FA visits to GPs drops significantly. The method is simple and repeatable. However, without a control group of GPs who have not received such information, it is impossible to assess if the intervention was the only circumstance affecting the reduction in FA consultation rates.

## Background

There is no generally accepted definition of a frequent attender (FA). Most studies have used the number of visits per year as the criterion, but the specific number chosen to define a frequent attender varies widely, from 5 visits per year at one end of the range to 20 visits at the other [1–7]. There are studies where FAs are defined as patients who have one-year attendance rates, adjusted for age and gender, above the 90th percentile [8, 9]. In Finnish studies, numerical definitions varying from at 8 to 11 GP visits per year have been generally used [1–4, 10].

The problem of frequent attenders has been studied for more than 60 years. Backett et al. reported in 1954 that 16% of patients made ten or more visits per year to general practitioners (GPs), and that these patients represented 52% of GPs’ workload [11]. Frequent consulters are a small proportion of all GPS’ patients but account for a disproportionate number of consultations [7]. Vedsted and Christiansen conducted a literature review in 2005, which found that the top 10% of attenders accounted for 30–50% of all GP contacts [12]. It is entirely acceptable to spend a lot of healthcare resources on patients whose condition demands it, but certain FAs can create unnecessary and unwelcome work and cause frustration for GPs [13].

Research by Heywood et al. (1998) found that FAs received many more prescriptions and were referred to hospital much often that other patients [14]; it is impossible to establish whether these treatment decisions were warranted by the condition of the patients concerned, or whether they represented poor use of healthcare resources. It has been estimated that a decrease of one visit per FA patient per year would decrease the average workload of a GP by 1 % [14]. In Finland, the average frequency of visits to a GP decreased from 1.92 visits to 1.56 visit per inhabitant per year in the period from 2001 to 2010. One FA makes 10 or more visits to a GP per year, so the difference is considerable.

Systematic reviews indicate that FAs often have chronic diseases or other chronic physical or mental problems [4, 12, 15–17], and that they may also have long-lasting somatization and many concomitant disorders [10, 18]. The majority of FAs are elderly females [3, 19, 20]. FAs’ socioeconomic status is usually low and they use many social services [12]. Other characteristics are a body mass index over 30, fear of death, low alcohol intake, low satisfaction with healthcare services and irritable bowel syndrome [3].

Studies have been conducted to investigate what kind of interventions might reduce FAs’ consultation rates. Research by Bellón et al. showed that intervention with GPs can be effective; in their study, three GPs received 15 h’ intervention training which incorporated biopsychosocial, organizational and rational approaches [21]. Jiwa tried to reduce Fas’ consultation rates by giving GPs summarized notes on their Fas’ medical histories which they could refer to during consultations but this intervention was not successful [22].

The present study was conducted in the city of Turku, which is the sixth largest city in Finland, with 175,000 to 178,000 inhabitants during the years of the study (2001–2010). Turku is an industrial and university city, with an immigrant population of about 8 % and an age distribution similar to most industrialized countries. GPs have a capitation-based contract, where each GP is responsible for about 1500–2700 inhabitants, with a mean of 2312 inhabitants per GP in 2010. Usually, patients first call a nurse, who assesses the type of treatment needed and, if necessary, arranges an appointment with the allocated GP. In this Finnish primary care model, nurses thus control access to GPs to some extent, and as a result FAs do not meet their GPs as often as in many other countries. The duration of GP consultations varies from 10 to 45 min.

The first aim of this study was to establish the total number of FA patients, and how many visits FAs made to the healthcare centres and primary care emergency clinic in Turku in 2001–2010. The second aim was to explore how administrative information provided to each GP about his/her FAs (names and number of visits for each FA in the preceding year) affects frequency of attendance. The hypothesis was that this simple intervention reduces frequent attendance by drawing the attention of GPs to the issue. Thirdly, for the years 2001–2010, we compared the overall workload of the GPs in the study with the workload arising specifically from consultations with FAs. We also asked the GPs to draw up treatment plans for their patients listed as FAs in 2004, and we checked later to see how many plans had in fact been made.

## Methods

The research design in this study is a registry-based cohort study. We used the developmental work research method; this model proceeds from evaluation of current action, to model and analysis of novel courses of action, to implementation and final assessment of the new courses of action [23]. In this study, FAs are defined as patients with 10 or more face-to-face visits to a GP during 1 year. The study population was formed by all patients who visited a GP ten times or more per year. The study data were retrieved from the electronic patient record system (Pegasos®) of the city of Turku, where the research was conducted. The electronic patient record system was used by every GP in all of the 11 public healthcare centres in the city and in the primary care emergency clinic during the whole period of 2001–2010. Data were collected on all face-to-face visits to GPs, both in the healthcare centres and the emergency clinic.

In 2002–2005, the chief medical officer of the city of Turku held three short administrative information sessions during regular management meetings with all GPs. In the 2002 session, all the GPs received personalized information about the number of FAs identified in their own patient register for the previous calendar year (2001). The same procedure was repeated in 2003 concerning the data for 2002. In 2005, the list of FAs was updated again according to data covering the year 2004, and the information was passed to GPs as before. In addition, in the 2005 session, the chief medical officer asked the GPs to make treatment plans for the FAs on the 2004 list.

In 2006–2010 the data of the number of FA visits to GPs were collected but in this period, the information was not passed on to GPs. To estimate the impact of providing GPs with administrative information on FAs, we compared the number of FAs and the total number of FA visits in the starting year (2001) with the corresponding figures in 2002–2006. The results are expressed as relative risks (RR) with 95% confidence intervals (95% CI). Then we analyzed the number of FAs and total FA visits in 2007–2010, i.e., over a period when the GPs had not received personalized information about their FAs. Finally, we compared those figures with the ones of 2006. The year 2006 was chosen as the comparison year because the final administrative information session for the GPs had been held in 2005.

The records of all patients listed as FAs in 2004 were checked during the follow-up period to see whether treatment plans had been made for them by their GPS. The number of treatment plans made was recorded.

Information on the number of GPs in 2001–2010, the number of days worked by each GP and the number of individual patients treated per workday was retrieved form the electronic patient record system.

The changes in the number of FA visits as a proportion of all visits to GPs were analyzed using Poisson’s regression analysis over the whole period of 2001 to 2010. The natural logarithm of number of all visits to GPs’ surgeries was used as an offset parameter in the Poisson model. The results were expressed as risk ratios (RR) with 95% confidence intervals (95% CI). *P*-values of less than 0.05 were considered statistically significant. Statistical analyses were carried out using the SAS system for Windows, Version 9.2 (SAS Institute Inc. Cary, NC, USA).

## Results

All GPs in this study were employed by the city of Turku. They were working in 11 primary healthcare centres in different parts of the city, and in the primary care emergency clinic. The number of full-time tenured GPs increased from 71 in 2001, to 74 in 2006 and 77 in 2010. In 2003, 2005, 2006 and 2008, the number of tenured GPs remained the same as in the previous year. In addition, a large number of locums were employed every year to cover for GPs on annual leave, study leave, and other leaves of absence; these locums were employed for different lengths of time and many worked part-time. Thus, during the years 2001–2010, an average of 135.6 individual GPs per year were employed by the city. Each GP worked 109 days per year on average and treated an average of 1003 individual patients annually.

During the years 2001–2010, a total of 1,816,457 face-to-face visits were made to the GPs, of which 166,059 visits were made by FA patients. The visits of the FA patients accounted for 9.1% (mean value) of all GP visits in primary healthcare. At the same time, FAs represented only 1.8% (mean value) of all patients (Table 1). The average number of individual patients was 73,096 per year, and of FA patients 1327 per year. FAs made on average 16,606 visits per year during the period covered by this study (Table 1).

**Table 1:** Total number of visits, number of FA visits, and proportion of FAs visits in 2001–2010

Year	Number of all visits	Number of FA visits	Proportion of FA visits of all visits %	Number of patients	Number of FAs	Proportion of FAs of all patients %
2001	194,217	17,627	9.1	75,560	1415	1.9
2002	186,341	16,898	9.1	73,930	1332	1.8
2003	183,184	15,876	8.7	72,302	1292	1.8
2004	180,007	15,886	8.8	71,840	1287	1.8
2005	180,380	16,140	8.9	73,185	1301	1.8
2006	178,987	15,276	8.5	73,208	1241	1.7
2007	179,361	16,620	9.3	72,370	1330	1.8
2008	182,394	17,393	9.5	73,860	1380	1.9
2009	174,486	17,104	9.8	71,968	1338	1.9
2010	177,100	17,239	9.7	72,738	1349	1.8

In 2001, the total number of FA patients was 1415, or 1.9% of all patients who used public healthcare services provided by GPs that year. The number of FAs varied between 1241 and 1415 during the subsequent 9 years (to 2010). The annual mean number of visits to a GP per FA varied between 12.3 and 12.8. The total annual number of FA patient visits went down from 17,627 in 2001, to 15,276 in 2006, although there was a reversal in the downward trend in 2005, when patient visits rose to 16,140 from 15,886 in 2004. FA visits as a proportion of all GP visits varied between 8.5 and 9.8%. Treatment plans were made for 73 of the patients listed as FAs in 2004, i.e. for only 5.9% of all FA patients that year.

At the outset in 2001, FA visits as a proportion of all visits to GPs was 9.1%. The GPs received information on their FAs between 2002 and 2005, and after these interventions, FA visits as a proportion of all visits to GPs decreased significantly in 2003 and even more so in 2006, compared with the situation in 2001. FA visits in 2003 had decreased from 9.1% (2001) to 8.7%, and in 2006 from 9.1% (2001) to 8.5% (*p* < 0.0001 for both comparisons). The number of FA visits was smallest in 2006, when it stood at 15276 visits (Table 1).

During the follow-up period 2007–2010, when there was no administrative information about FAs given to the GPs, there was a significant (*p* < 0.0001, Table 2) increase in the proportion of FA visits when compared with the proportion of FA visits in 2006.

**Table 2:** Changes in the proportion of FA visits of all visits over the period 2001–2010

Years compared	RR	95% CI		*p*-value*
2002 with 2001	0.99	0.978	1.03	0.9379
2003 with 2001	0.95	0.93	0.98	< 0.0001
2004 with 2001	0.97	0.95	0.99	0.0104
2005 with 2001	0.99	0.97	1.01	0.1918
2006 with 2001	0.94	0.92	0.96	< 0.0001
2007 with 2006	1.09	1.06	1.11	< 0.0001
2008 with 2006	1.12	1.09	1.14	< 0.0001
2009 with 2006	1.15	1.12	1.17	< 0.0001
2010 with 2006	1.14	1.12	1.17	< 0.0001

Intervention in the form of individualized information to GPs on frequent attendance was provided in 2002, 2003, and 2005. An RR of more than 1.00 signifies that there was an increase in FA visits

*RR* Relative Risk, *CI* Confidence Interval

* Statistical significance between years; Poisson’s regression analysis

Between 2001 and 2006, the number of FA visits decreased by 2351 visits. The average number of all patient consultations per GP in 2006 was 1234.4, so this reduction corresponded to the annual workload of 2 GPs. There was a very low response to our request for GPs to draw up treatment plans for their patients listed as FAs in 2004; plans were made for only 73 patients, less than 6% of FAs in that year.

## Discussion

In the city where this study was conducted, FAs comprised 1.8% of all patients; this percentage is consistent with other studies which report proportions ranging from 1.7–4.7% [1, 6]. There are studies in which the proportion of FAs was found to be considerably higher - from 10.6% to as much as 15.4% [14, 24]. This can be explained by differences in defining an FA, and differences between healthcare systems. During the first decade of this millennium, the total number of GP consultations decreased in the city in question, and similar decreases happened over the same period elsewhere in Finland, too. Although the reasons for this phenomenon are unclear, it is possible that the overall number of GP consultations has gone down because individual consultations tend to be longer now than before, as patients’ problems become more complex and more time-consuming to deal with [25].

In 2002, 2003 and 2005, GPs in the city’s primary healthcare system received administrative information about their FA patients: their names and number of GP visits made by each one during the preceding year. This rather simple procedure seemed to reduce the number of FA patients’ visits to a GP. Compared with the figure for 2001 (17,627 FA consultations), the decrease in the annual consultation rate of FA patients was significant in both 2003 and 2006. When the administrative information was no longer provided, the FA consultation rate per year increased again, as seen by comparing the figure for 2006 with the figures for the four subsequent years (2007–2010). A systematic literature review conducted in 2008 concluded that there is no evidence that FAs’ utilization of healthcare services can be reduced [26]. The results of our study, however, are more optimistic; the simple procedure of providing administrative information directly to GPs did seem to reduce FAs’ consultation rates. In fact, the reduction in the consultation rates of FA patients from 2001 to 2006 corresponds to the annual workload of 2 GPs. This finding is in line with the conclusions of Heywood et al. [14].

The information about FAs was provided once annually in 2002, 2003 and 2005, to all GPs who were working in the city of Turku at the time of the information session, but the fluctuation in the number of individual GPs from year to year, and the low number of full-time GPs might influence the findings. The number of the full-time GPs was unchanged in 2003, 2005, 2006 and 2008. Locums are hardly the explanation for the decrease in FAs’ visits in 2003 and 2006. In 2004, on the other hand, the number of full-time GPs changed which may partly explain why there was no reduction in FA patients’ visit rates in 2005. Furthermore, no administrative information on FAs was provided in 2004, which may also have contributed to the slight reversal in 2005 in the overall downward trend. During this decade, the number of tenured GPs increased by six, and the number of locums varied. The effects of the fluctuations in the number of full-time GPs and the large number of medical officer locums are not known and cannot be analysed in this study setting.

The administrative information on FA patients was provided in the course of regular team meetings between the GPs and the chief medical officer; thus there was no parallel group of uninformed GPs to act as a control group. The method used was very simple. In the study of Bellón et al., GPs received 15 h’ training about the issue of FA-patients, and this intervention, too, yielded a significant reduction in the frequency of FA consultations [21]. Our intervention to GPs was much simpler and considerably less time-consuming, as it took place once a year in the course of normal meeting.

GPs made only few treatment plans to FAs. It is impossible to say whether treatment plans would be helpful in improving the health or reducing the visit rate of FA patients.

The electronic patient record system used in the health care centres involved in this study does not have useful tools for enabling the GPs to monitor their work. This might be one reason why providing administrative information in face-to-face sessions turned out to be important. For example, the electronic patient record system does not allow for serial numbering of patient contacts during the year; such a feature would enable GPs to recognize instantly the patients making frequent visits.

The strength of the present study is the large amount of data on GP visits during 2001–2010, covering 1,816,457 appointments, including 166,059 appointments with FAs. Also, providing GPs with the administrative information was simple, quick, and easily repeated procedure. A weakness may be the fact that the administrative information provided was not methodologically standardized; it was incorporated into managerial routine. Another weakness was that we had no possibility to use a control group of GPs who had not received information on their FA patients; this methodological drawback arose from the realities of everyday management. The results need to be confirmed by further comparative studies.

## Conclusion

Providing administrative information to GPs about their frequently attending patients, including their names and the frequency of their visits, yielded only a modest overall reduction in the number of frequent attenders over the period covered by the study. However, in two of the years (2003 and 2006), there were significant reductions in the total number of visits of FA patients, even though the numbers of FA patients themselves decreased only slightly**.** It thus seems that this simple intervention can reduce the annual number of visits by FAs to public healthcare centres, rate of frequent attenders was significantly reduced. It seems that such administrative information can reduce the number of annual visits by FA-patients in public healthcare centres, where GPs are working as a team and are led by one chief medical physician. To our knowledge, this is the first study to show that this kind of simple administrative information given by the chief medical officer to the GPs can significantly reduce the consultation rate of frequently attending patients.
